# An informational video for informed consent improves patient comprehension before total hip replacement- a randomized controlled trial

**DOI:** 10.1007/s00264-025-06503-6

**Published:** 2025-04-02

**Authors:** Sebastian von Hertzberg-Boelch, Konrad Fuchs, Johanna Schubring, Dominik Rak, Kilian List, Konstantin Horas, Axel Jakuscheit, Maximilian Rudert

**Affiliations:** 1 LVR Clinic for Orthopaedics Viersen, Viersen, Germany; 2 Department of Orthopaedic Surgery, Julius-Maximilian University Wuerzburg, Wuerzburg, Germany; 3 Department for Trauma Surgery, University Hospital Wuerzburg, Wuerzburg, Germany

**Keywords:** Informed consent, Total hip arthroplasty, Video, Shared decision

## Abstract

**Purpose:**

Effective patient comprehension is critical for informed consent, particularly in Total Hip Arthroplasty (THA), a globally prevalent procedure. This study evaluates the efficacy of an informational video to improve the patients' understanding, self-perceived knowledge, and emotional comfort in the context of THA informed consent. This randomized controlled trial investigates the impact of an additional informational video on (I) the patients' understanding, (II) self-precepted knowledge and (III) emotional comfort during the informed consent process for THA.

**Methods:**

Participants were randomized to receive either the standard informed consent procedure or the standard procedure supplemented with an informational video. The effect of the video was tested with post-consent questionnaires.

**Results:**

The informational video significantly (*p* = 0.014) improved the patients' understanding from 78.6% to 86.5%. Self-precepted knowledge and Emotional comfort was not effected by the video (*p* = 0.986; *p* = 0.333).

**Conclusions:**

The informational video significantly improved patient comprehension during the informed consent process before THA.

**Supplementary Information:**

The online version contains supplementary material available at 10.1007/s00264-025-06503-6.

## Introduction

### Background

The patients' understanding of a procedure is regarded as a fundamental prerequisite for shared decision making and informed consent. Total Hip Arthroplasty (THA) resolves pain and improves quality of life making it a highly effective procedure for hip arthritis [1]. Therefore, an immense increase of THA procedures is globally anticipated [2].

To date, there are no studies, evaluating what is the best approach to informed consent for THA [3]. More than 20 years ago Langdon et al. demonstrated, that a short, standardized information form comprising less than two pages improves the patients' understanding of the upcoming THA procedure [4]. To date, the use of standardized information forms has become routine for the information process [5]. In contrast to the short forms used by Langdon et al. the currently available information forms are not only designed to educate and inform the patient, but also to document that the legal obligations for the surgeon have been fulfilled [6]. Particularly for THA, these forms have become highly complex and can comprise up to six or even more pages. Unsurprisingly, that these forms can be overwhelming and insufficient for informed consent due to their complexity. Additionally, the long sequence of bullet-pointed complications has induced anxiety or even ignorance [3]. Irrespectively of the procedure, the content of these forms is commonly explained to the patient by a physician [7]. However, this approach to informed consent is characterized by variable effectiveness, which, amongst others, is rooted in insufficient patient understanding, a lack of basic information from the consent form and an ineffective physician patient communication [8].

### Rationale

Audio-visual means for patient information are nowadays readily available and have demonstrated great potential to improve the patients' understanding during the information process [7, 9]. Clearly, the information process to informed consent on THA and its aftercare has to be adjusted to the patient's requirements and his individual capacity [6]. However, the vast majority of the content given during the information is the same for all the patients—particularly in highly specialized THA centres, which already now, perform thousands of THA implantations per year [10].

The study hypothesizes that audio-visual aids can significantly improve patient comprehension. It aims to compare the effectiveness of a standard informed consent process against one, augmented with an informational video, focusing on (I) the patients' understanding, (II) self-precepted knowledge, and (III) emotional comfort with the procedure.

## Methods

### Ethics

This prospective randomized controlled single centre study was approved by the institution's ethics committee (Ethics Committee of the University of Wuerzburg, Germany, IRB number 257/20) as an investigator initiated study and registered at the German Registry for clinical studies (trial number DRKS00025874). No grants were received by the investigators in the context of this study.

### Trial set up

Participants were recruited from patients scheduled for THA at the study institution. All patients gave written consent to participate in the study. Informed consent before THA is accomplished and signed at the day of admission, usually the day before surgery. Therefore the patients receive a standardized form (DE617288 distributed by Thieme Compliance GmbH, Erlangen, Germany) which is explained by a physician. For the trial the control group underwent this routine informed consent process. In addition to this, the patients in the intervention group, watched a specially designed, 18 min informational video. The video was accessed via a link sent by personal e-mail one to three days prior to admission. This informational video had been developed by the authors in collaboration with a medical informational video company, (medudoc, Berlin, Germany) (Content for informed consent in the video is provided in the appendix). The physicians conducting informed consent during admission were not aware of the group assignments.

### Outcome measures

The effect of the video was measured with three Questionnaires. The questionnaires assessed patient knowledge (Q1), self-precepted knowledge (Q2) and emotional comfort about the procedure (Q3). The questionnaires were developed by three high volume THA surgeons. For Q1, the different aspects of informed consent were considered as described by Shah et al. [6] by the following sections: "explanation (of the procedure)", "participation (in decision making)", "alternatives (to the procedure)" and "risks (of the procedure)". These sections comprised 12, eight, eight and 14 statements, respectively. The patients were asked to categorize these statements as "correct" or "false". In Q2 the patients were asked to rate eight statements on self-precepted knowledge on the afore mentioned sections as "fully understood", "understood most", "understood in parts", "understood not enough" or "not understood". In Q3 patients were ask to assess their level of anxiety regarding the procedure, rate their satisfaction with the informed consent process and evaluate the quality of preparation for the procedure on a scale of five categories.

The questionnaires were handed to the patients directly after informed consent was completed. The patients received no help with the questionnaires. Patients from the intervention group had to confirm, that they had watched the informational video.

### Power analysis

The primary aim of the present randomized study was to compare two groups of patients (additional video-based information vs. no additional information) regarding their understanding of the planned procedure assessed with the questionnaire I, from which a score was calculated (scale 0–42 points). It was assumed that the two groups would differ by at least 5 points in the score, with a standard deviation of the score value of σ = 5. This difference can be demonstrated with a total sample size of *n* = 34 patients (17 per group) at a significance level of 5% and with a power of 80%.

### Patient inclusion and randomization

Study participants were selected from individuals scheduled for Total Hip Arthroplasty (THA) at their initial consultation. Eligibility criteria included planning for THA according to standard procedures, an age range of 18–75, absence of dementia, primary osteoarthritis diagnosis, and proficiency in German (both speaking and reading), along with the ability to see, hear, and provide informed consent. Randomization was performed using sealed envelopes. Patients with missing answers in QI were excluded from analysis. Patient inclusion was conducted until both groups comprised at least 17 participants with complete answers in QI.

### Statistics

A t-test for independent samples was used to compare metric variables. A Chi-Square test was used to test for correlation of categorial and nominal variables. Statistics were conducted with SPSS. Statistics are displayed as Mean and standard deviation (SD).

## Results

### Patients

74 patients had to be included until the intervention group and the control group met the required number of participants in both groups with correctly completed questionnaires I. Thus, the intervention group comprised of 20 and the control group of 17 patients.

### Primary outcome

#### Patients' understanding (Q1)

**Fig. 1:**
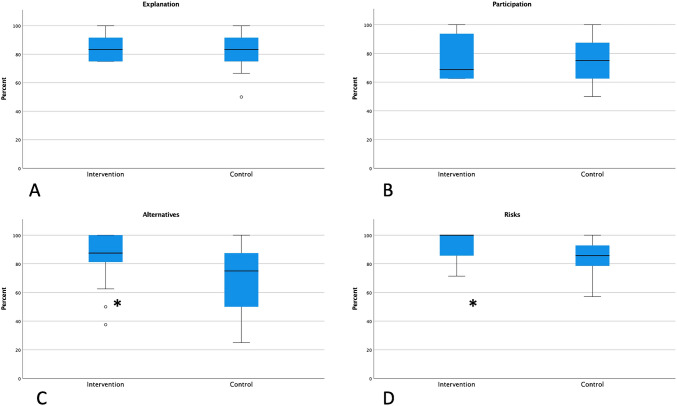
Percent of correct answers for the intervention and the control grouped by subcategories of informed consent (**A**) Explanation; **B** Participation; **C** Alternatives; **D** Risks; *: significant difference in C with *p* = 0.009 and D with *p* = 0.016

The rate of correct answers in the intervention group was 86.5% compared to 78.6%. This difference was significant (*p* = 0.014). The differences for the subsections are shown in Fig. 1.

### Secondary outcome

#### Self-precepted knowledge (Q2)

**Fig. 2:**
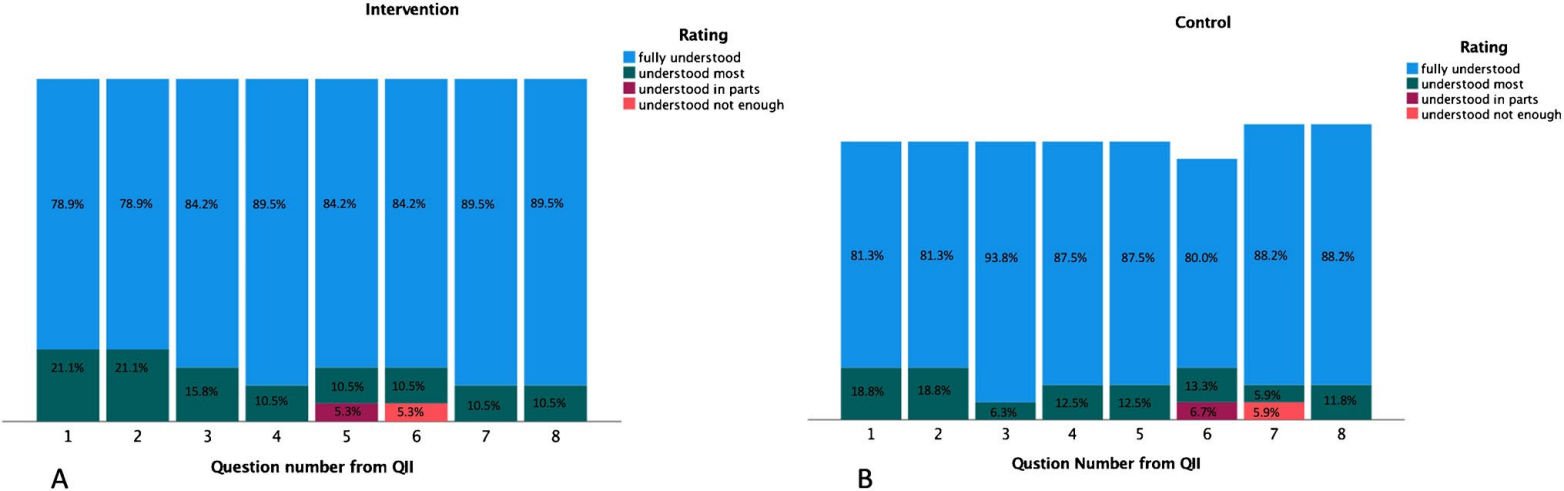
Distribution of ratings on self-precepted knowledge for each question in questionnaire II for the intervention (**A**) and the control (**B**)

Self-precepted knowledge was rated as "fully understood" in 85.9% in the intervention group and 86.0% in the control group, respectively. The video had no significant effect on self-precepted knowledge (*p* = 0.986). Figure 2 depicts the distributions of ratings.

#### Emotional comfort (Q3)

The video had no significant effect on the ratings for emotional comfort (QIII) (*p* = 0.333).

**Fig. 3:**
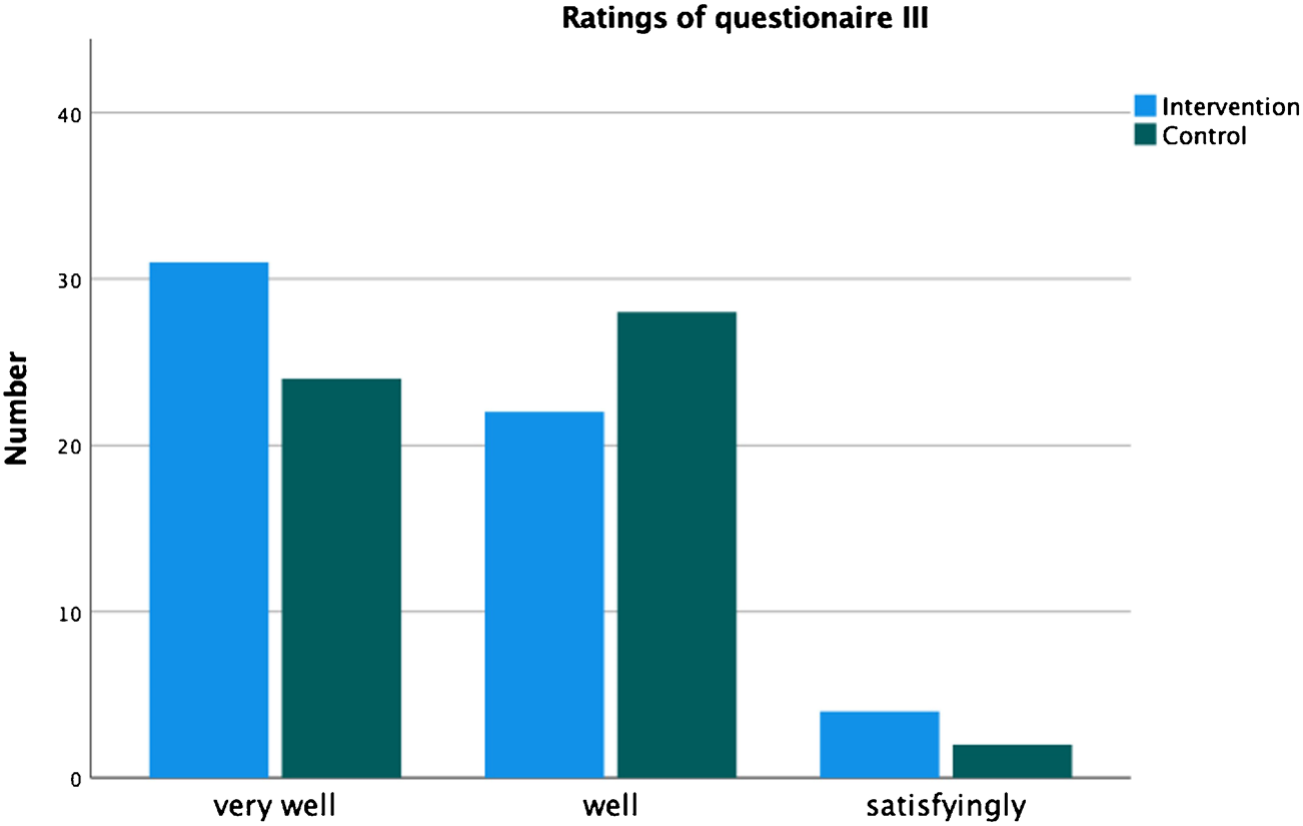
Answers given to the three questions in Q III. The answer possibilities "sufficiently" and "insufficiently" are not shown, because they were not selected

Distribution of rating are shown in Fig. 3.

## Discussion

The information provided during the informed consent process empowers the patient to autonomously decide and authorize the surgeon to perform the THA procedure [5, 6, 11]. This information is most commonly explained by a physician to the patient with the help of standardized forms. However, this approach does not guarantee sufficient understanding for shared decision making and informed consent [8]. Compared to this routine approach, the current study demonstrates, that patients have a better understanding of the THA procedure, if an informational video is incorporated into the information process. Additionally, it is also important to state that better understanding of the procedure does not come at the cost of emotional comfort with the upcoming THA implantation.

These results are in accordance with the review by Farell et al. published already in 2014, who analyzed 29 randomized controlled trials and concluded, that audio-visual information aids improve patient understanding [9]. The newer review by Glaser et al. from 2020 summarized the effect of audio-visual information aids in comparison to a physician patient discussion with the help of standardized forms. Although compared to Farell et al. the effect was not as distinct, audio-visual aids improved patient understanding in 65% of the studies [7]. None of the studies analyzed in these reviews focused particularly on patients undergoing THA. A large study with 151 patients by Johnson et al. investigated the effect of audio-visual aids for informed consent before total knee replacement. In this three-armed trial the control arm was informed by iMedConsent and a customized written handout, the first study arm received an extra information through a video and the second study arm an additional video plus formal education. However, no effect of the different approaches was observed [12]. In contrast to the trial by Johnson et al. the current two armed trial shows significant improvement of the patients' understanding by more than 10%. It has to be emphasized, that the aspects "alternatives" and "risks" play a particularly crucial role for informed consent [3, 5, 13]. For these specific aspects, the informational video had clearly pronounced effects: Understanding of "alternatives" and of "risks" was improved significantly by 17.0% and by 10.3%, respectively. Concerns of the physician to induce anxiety or doubts about the upcoming THA are possible explanations for the strong effect of the video [3].

In contrast to the common setting, in which the explanation of the content of the standardized form is delegated to residents with little surgical experience or not having undergone structured teaching and training on patient information [14], experienced THA surgeons agreed on how to provide this content by video. Thus, it might also be the insufficiency of the physician patient discussions and the complexity of the form sheets that were no able to provide the relevant information as reliable as the video.

Potential bias exists because validated questionnaires for patient understanding are not available and the same surgeons also developed the questionnaires to measure the effect.

However, broad consent on what patient understanding is desirable, has to be sought by the orthopaedic community, no only for medical but particularly for medicolegal reasons [15]. In this context, an informational video does not only improve but also standardizes the information process to informed consent. This evolution could counteract the increasing availability of misinformation transported particularly through social media [16].

To date, doctors are advised, to give the patients "all reasonable help and support to make a decision and to inform patients about reasonable alternatives" [13]. Based on the herein presented findings, surgeons providing THA should be encouraged to incorporate informational videos in the informed consent process.

## Conclusion

The utilization of informational videos in the informed consent process prior to Total Hip Arthroplasty (THA) has the potential to enhance this process. Continued research in this area could lead to more efficient use of personnel resources, increased patient satisfaction post-THA, and possibly a decrease in the incidence of related legal actions.

## Supplementary Information

Below is the link to the electronic supplementary material.

Supplementary file1 (DOCX 17 kb)

Supplementary file2 (DOCX 14 kb)

Supplementary file3 (DOCX 14 kb)

Supplementary file4 (DOCX 21 kb)

## Data Availability

No datasets were generated or analysed during the current study.
