# Supplementary material for: An informational video for informed consent improves patient comprehension before total hip replacement- a randomized controlled trial
Source: Int Orthop. 2025 Apr 2;49(6):1303–8. doi: 10.1007/s00264-025-06503-6 (PMC12075017; doi:10.1007/s00264-025-06503-6)
Supplement: Supplementary file 1 — Supplementary file1 (DOCX 17 kb) [file 264_2025_6503_MOESM1_ESM.docx]

Questionnaire I

After your information session, please answer some questions about the content you just heard. For each statement below, please decide which you believe is true or not. Place a check mark next to "applies" or "does not apply" for each statement

1) How is the surgery performed?

| applies | does not apply |  |
| --- | --- | --- |
| € | € | The approach to the hip joint is done through a particularly muscle-sparing technique |
|  |  |  |
| € | € | For the procedure, the head of the thigh bone needs to be removed. |
|  |  |  |
| € | € | The entire thigh bone needs to be removed for the procedure |
|  |  |  |
| € | € | For the procedure, a short skin incision is made in the front-lateral abdominal region |

2) Please evaluate the following statements regarding the reasons for the upcoming surgery?

| applies | does not apply |  |
| --- | --- | --- |
| € | € | The procedure was recommended due to the physical limitations that I expressed to the doctors |
|  |  |  |
| € | € | The findings of the X-ray do not necessarily require the procedure itself. |
|  |  |  |
| € | € | The radiological changes always correlate with the extent of hip discomfort |
|  |  |  |
| € | € | The radiological changes do not always correlate with the extent of hip discomfort |

3) What are the goals of the planned procedure?

| applies | does not apply |  |
| --- | --- | --- |
| € | € | Improvement of mobility. |
|  |  |  |
| € | € | Reduction of pain in and around the hip joint. |
|  |  |  |
| € | € | To prolong the intake of pain medication |
|  |  |  |
| € | € | To shorten the maximum walking distance. |

4) Blood-thinning medications

| applies | does not apply |  |
| --- | --- | --- |
| € | € | They should never be stopped before the surgery. |
|  |  |  |
| € | € | They should be stopped in consultation with the treating physician well in advance. |
|  |  |  |
| € | € | They should be stopped at least one month in advance |
|  |  |  |
| € | € | They do not play a role in the upcoming surgery. |

5) Are there complications, how are they dealt with, and how likely are they?

| applies | does not apply |  |
| --- | --- | --- |
| € | € | Complications can occur but are rare. |
|  |  |  |
| € | € | Possible complications are addressed during the operation with specific measures. |
|  |  |  |
| € | € | The post-operative check-ups are conducted to detect complications early |
|  |  |  |
| € | € | Possible complications are addressed during the hospital stay with specific measures |

6) Possible postoperative complications include:

| applies | does not apply |  |
| --- | --- | --- |
| € | € | Leg length discrepancy |
|  |  |  |
| € | € | Infection |
|  |  |  |
| € | € | Dislocation of the prosthesis |
|  |  |  |
| € | € | Nerve injury |
|  |  |  |
| € | € | Allergic reactions |
|  |  |  |
| € | € | Thrombosis |

7) After the surgery

| applies | does not apply |  |
| --- | --- | --- |
| € | € | a 48-hour bed rest is always recommended. |
|  |  |  |
| € | € | Walking and getting up exercises are not recommended. |
|  |  |  |
| € | € | Early mobilization is aimed for in uncomplicated cases. |
|  |  |  |
| € | € | Generally, independent stair climbing is possible only after one month. |

8) What aftercare will be required following the procedure?

| applies | does not apply |  |
| --- | --- | --- |
| € | € | Beyond the hospital stay, abdominal injections will need to be administered for a certain period. |
|  |  |  |
| € | € | Blood-thinning medications should be stopped immediately after discharge. |
|  |  |  |
| € | € | Regular check-ups are recommended after hospital discharge. |
|  |  |  |
| € | € | The plaster is never changed during the hospital stay. |

9) What alternatives exist to the planned procedure?

| applies | does not apply |  |
| --- | --- | --- |
| € | € | Physiotherapy. |
|  |  |  |
| € | € | There are no alternatives. |
|  |  |  |
| € | € | Weight gain. |
|  |  |  |
| € | € | Pain medication. |

10) What is the course of the condition without the planned procedure?

| applies | does not apply |  |
| --- | --- | --- |
| € | € | Despite ongoing symptoms and potential limitations, people with hip joint arthritis can continue to live |
|  |  |  |
| € | € | Improvement of symptoms is unlikely. |
|  |  |  |
| € | € | In the course of the condition, mobility improves. |
|  |  |  |
| € | € | Generally, one becomes symptom-free again after several years. |
