# Supplementary material for: An informational video for informed consent improves patient comprehension before total hip replacement- a randomized controlled trial
Source: Int Orthop. 2025 Apr 2;49(6):1303–8. doi: 10.1007/s00264-025-06503-6 (PMC12075017; doi:10.1007/s00264-025-06503-6)
Supplement: Supplementary file 2 — Supplementary file2 (DOCX 14 kb) [file 264_2025_6503_MOESM2_ESM.docx]

Questionnaire II

Please estimate and mark how well you feel informed regarding questions 1 to 8.

1) How is the procedure performed?

 fully *understood*   *understood most*  *understood in parts*

 *understood not enough*  *not understood*

2) What are the reasons for the planned procedure?

 fully *understood*   *understood most*  *understood in parts*

 *understood not enough*  *not understood*

3) What are the goals of the planned procedure?

 fully *understood*   *understood most*  *understood in parts*

 *understood not enough*  *not understood*

4) Are there complications, and how likely are they?

 fully *understood*   *understood most*  *understood in parts*

 *understood not enough*  *not understood*

5) What complications can occur?

 fully *understood*   *understood most*  *understood in parts*

 *understood not enough*  *not understood*

6) How is the course of the condition without the planned procedure?

 fully *understood*   *understood most*  *understood in parts*

 *understood not enough*  *not understood*

7) What alternatives to the planned procedure exist?

 fully *understood*   *understood most*  *understood in parts*

 *understood not enough*  *not understood*

8) What aftercare will be required following the procedure?

 fully *understood*   *understood most*  *understood in parts*

 *understood not enough*  *not understood*
