# Supplementary material for: An informational video for informed consent improves patient comprehension before total hip replacement- a randomized controlled trial
Source: Int Orthop. 2025 Apr 2;49(6):1303–8. doi: 10.1007/s00264-025-06503-6 (PMC12075017; doi:10.1007/s00264-025-06503-6)
Supplement: Supplementary file 3 — Supplementary file3 (DOCX 14 kb) [file 264_2025_6503_MOESM3_ESM.docx]

Questionnaire III

1) How well did you feel prepared for the surgery through the information session? (Please check only once)

 *very well*  *well*  *satisfyingly*  *sufficiently*

 *insufficiently*

2) How satisfied were you with the information session? (Please check only once)

 *very well*  *well*  *satisfyingly*  *sufficiently*

 *insufficiently*

3) How well did the information session reduce your anxiety of the surgery? (Please check only once)

 *very well/no anxiety*  *well /slight anxiety but optimistic*  *satisfyingly/hesitating but convinced*  *sufficiently/hesitating*

 *insufficiently/reluctant*
