# Supplementary material for: An informational video for informed consent improves patient comprehension before total hip replacement- a randomized controlled trial
Source: Int Orthop. 2025 Apr 2;49(6):1303–8. doi: 10.1007/s00264-025-06503-6 (PMC12075017; doi:10.1007/s00264-025-06503-6)
Supplement: Supplementary file 4 — Supplementary file4 (DOCX 21 kb) [file 264_2025_6503_MOESM4_ESM.docx]

Spoken text in the informational video

Introduction

Welcome to your briefing on the process of your hip operation. Our clinic team typically refers to it as "TEP", which stands for Total Endoprosthesis, meaning the hip prosthesis.

Based on your health condition, we will explain the following points for your operation: Reasons for the operation; Preparation; Procedure of the operation; Risks during the operation; Risks after the operation; Postoperative course and behavior after the operation.

As a specialized clinic in endoprosthetics, HIP-TEP implantation is a routine procedure for us. However, we are aware that the procedure is associated with many uncertainties for the patient, and that the patient entrusts themselves to our clinic team. Below, we would like to explain to you why certain procedures are done and how you can participate in getting back on your feet as quickly as possible.

Indication and Alternatives o surgery

You are experiencing painful wear, known as arthritis, of the hip joint. Because your suffering is significant and you are having difficulty managing daily activities, the option of a hip prosthesis implantation has been presented to you. Due to the discomfort, you desire this operation.

The hip joint consists of two joint partners: the pelvis, or a specific part of the pelvis called the acetabulum, and the head of the thigh bone. Both joint partners, the acetabulum and the thigh bone's head, are covered with cartilage. The cause of your discomfort is the wear of the joint cartilage and, potentially, the bone underneath. This condition is known as hip joint arthritis. This arthritis can lead to pain when starting to walk, load-dependent pain such as when or after walking or standing, and sometimes even nocturnal pain. In addition to pain, arthritis often manifests as restricted movement of the hip joint

In the discussion with the doctor, possible treatment alternatives were explained to you. Treating arthritis is possible without surgery. For example, physical therapy or pain medication can alleviate symptoms. If non-surgical, conservative measures have been exhausted, consideration may be given to a total hip replacement. In cases of significant discomfort, surgery is a very good option. The goals of surgery are pain relief and improvement of daily function.

The timing of the operation primarily depends on the course of your symptoms and your level of discomfort. Therefore, hip replacement surgery is a planned procedure that you can prepare for. If you have any uncertainties, you can always contact your treating doctor or the patient management for assistance.

Admission

In the coming days, you will undergo pre-admission assessment. Here, you will have the opportunity to speak with doctors and nursing staff.

The pre-admission assessment is a final check-up before the operation. You will have discussions, examinations, and receive many documents to ensure that you are optimally prepared for the surgery not only from a medical perspective but also from a legal standpoint.

Prepare yourself for being hospitalized for several days. Depending on what you discuss with the social service, you may subsequently stay at a rehabilitation clinic. It would be particularly helpful to have comfortable and secure footwear and easy-to-wear clothing, such as tracksuits and bathrobes.

If you regularly take blood-thinning medications, you should stop them in advance of the procedure after consultation with your treating doctor.

As part of the preparation for the surgery, you will have a discussion with an orthopedic resident. During this discussion, the affected joint will be examined again, and if necessary, further investigations will be initiated. This is where you will receive information about the procedure and provide written consent for the surgery.

To prepare for the anesthesia procedure for the surgery, you will have a discussion with an anesthesiologist. It would be helpful if you bring along any previous medical records. During this discussion, you will receive information about the anesthesia procedure and provide written consent for it.

Operation Technique

The procedure can be performed under general anesthesia or spinal anesthesia. The anesthesiologist will discuss which method is recommended for your specific case during the preparation

A hip operation is a routine procedure that takes approximately 50 to 90 minutes. The choice of the endoprosthesis, as well as the type of anchoring, depends on your health condition, bone quality, and physical activity.

Based on a computer-assisted planning sketch, the size and position of each prosthesis component are individually tailored to you. Furthermore, as part of the surgical planning, the type of hip prosthesis to be used and the best type of fixation for you will be determined.

Your joint replacement will be inserted using a particularly muscle-sparing procedure known as a minimally invasive technique. This approach avoids a large skin incision and preserves the muscles, vessels, and nerves. As a result, you can usually fully bear weight on the operated side immediately after the surgery, accelerating the healing process.

The hip joint is exposed without damaging muscles, nerves, or tendons. The diseased femoral head is removed. Then, the damaged joint surface of the hip socket is prepared and replaced with an artificial hip socket. A prosthetic stem is inserted into the thigh bone. The artificial joint head is then placed onto the stem, and the hip is repositioned.

Hip joint prostheses are most commonly anchored without cement. In special situations, it can be advantageous to anchor the prosthesis with bone cement, a fast-hardening plastic. The decision for this is often made during the operation.

To achieve mobility between both joint parts of the prosthesis, special materials are used for hip prostheses. These materials are carefully matched to ensure that the artificial joint can function as long as possible.

You will be given special medications for your operation. These medications reduce the risk of bleeding during the procedure and decrease the likelihood of swelling after the operation.

These medications are tranexamic acid and dexamethasone. For this application, both drugs are used in what is known as "off-label use." Existing literature and our experience strongly support the use of these medications, provided there are no contraindications.

An X-ray will be taken in the operating room to check the position of the artificial joint and to definitively rule out any possible bone injuries.

At the end of the operation, the surgeon will thoroughly check your new joint for mobility and functionality. Afterwards, the wound will be closed and dressed.

The administration of anesthesia medications will be stopped. You will then gradually and carefully regain consciousness.

Risks during the operation

During the operation, complications occur very rarely. However, unwanted incidents can occur during the procedure. The risk of complications depends on the type of surgery, your health condition, and your risk profile

By anchoring the artificial hip joint, fractures in the pelvic and thigh areas may occur, although the risk is low. This complication can be quickly identified and treated through X-ray monitoring during the operation. In such cases, the operation may need to be extended, and postoperative care, such as partial weight-bearing, may need to be adjusted accordingly.

If there is a significant blood loss during the operation, for example due to a blood vessel injury, it may be necessary in rare cases for you to receive a blood transfusion.

In the area of the skin incision, there is a nerve responsible for sensation on the outer thigh. This nerve can potentially be injured during the operation. However, the likelihood of such nerve injury, which also supplies the surrounding muscles, is very low.

Despite the gentle surgical approach, soft tissues such as muscles and blood vessels can still be injured or damaged. However, the risk of tissue damage is very low.

Medication administration can lead to allergic reactions. We monitor your levels closely and can respond promptly if necessary.

Risks after the operation

In some cases, it may be necessary to slightly lengthen the leg during the operation. Usually, patients do not perceive this as bothersome after the operation. However, differences in length and axis deviation can cause discomfort. Symptoms that may arise can potentially be alleviated or entirely avoided through the use of orthotic shoe inserts and physiotherapy

After the placement of your artificial hip joint, wound healing issues can occur. To prevent this, your wound will be regularly monitored and cared for.

Rarely, there can be dislocation of the artificial joint, known as hip dislocation. In the worst case, this may require another surgery.

Collection of blood outside blood vessels known as hematoma can occurr. In most cases, it remains mild and does not cause pain. However, in exceptional cases, a repeat surgery may be necessary.

Pain after surgery is not unusual. It typically diminishes within a few weeks. Some initial discomfort when starting to move can persist for several weeks but usually resolves completely.

Despite preventive measures such as abdominal injections and compression stockings, there is a risk of vascular occlusion during and after hip surgery due to prolonged immobilization. This can lead to the formation of a blood clot, known as thrombosis. Such a blood clot can dislodge from its original location and block a blood vessel elsewhere in the body.

After hip surgery, bacterial colonization around the implanted hip prosthesis can occur. Depending on the severity of the infection, it can be treated with medications such as antibiotics or may require a repeat operation.

Hip prostheses have a long lifespan of 15 to 20 years, and in some cases even 25 years or more. However, wear and loosening of the prosthesis can occur at any time. If necessary, a hip prosthesis replacement may be required.

In isolated cases, material damage can occur after prosthetic anchoring. However, the materials used today typically have a long lifespan.

After the operation, signs of nerve damage, ranging from numbness to paralysis, can occur. Fortunately, most of these disturbances are not permanent and can resolve with appropriate treatment.

After joint replacement, an allergic reaction to components of the prosthesis can occur, potentially necessitating a revision surgery. However, implant allergy itself is highly debated in the medical community.It's important to inform your treating doctor about any allergies you have beforehand so that alternative materials can be considered if necessary.

During the hospital stay

After the procedure, you will be monitored and cared for in the recovery room. After a few hours, you will be transferred to an observation ward for further monitoring.

After your operation, you will be temporarily cared for in an intensive care unit (ICU). Here, your breathing and circulation will be monitored closely. You will remain under observation until you can be transferred to a regular hospital ward, typically within 6 to 24 hours. The ICU provides the opportunity to closely monitor and manage any pain that may arise immediately after the operation. During this time, you may be visited by your family members.

During your days on the regular hospital ward, you will be regularly visited by nursing and medical staff. Please inform us if you experience any pain after the operation. It is important to manage pain effectively not only for immediate comfort but also for the long-term success of your treatment.

Your hip prosthesis can and should be weight-bearing from the beginning. Early mobilization with movement exercises can help reduce the risk of complications.

In a typical course, you can start with initial standing and walking exercises supported by physiotherapy on the day of the operation. Upon discharge, usually within 3 to 5 days, patients can usually climb stairs independently. Physiotherapists are available to provide assistance as needed

Routine X-rays are typically taken around 2 to 3 days post-surgery to check the correct positioning of the prosthesis.

During your hospital stay, regular medical check-ups will be conducted to ensure that no complications arise post-surgery. These examinations will assess circulation, mobility, sensation, and pain. We rely on your feedback during these assessments. Please inform us of any concerns or unusual developments outside of these scheduled check-ups.

After Dismissal

Once your independence permits, we recommend discharge to your home. It's advisable to have someone pick you up or accompany you. Upon discharge, you will receive a certificate of incapacity to work for at least four weeks, depending on your occupation.

During your upcoming pre-admission appointment, we encourage you to ask any remaining questions you may have during the discussion about the operation or anesthesia procedure. This is an opportunity to clarify any concerns you may have before your admission.
